# A binary acceleration signal reduces overestimation in pedestrians’ visual time-to-collision estimation for accelerating vehicles

**DOI:** 10.1016/j.heliyon.2024.e27483

**Published:** 2024-03-06

**Authors:** Marlene Wessels, Daniel Oberfeld

**Affiliations:** Institute of Psychology, Section Experimental Psychology, Johannes Gutenberg-Universität Mainz, Wallstrasse 3, 55122, Mainz, Germany

**Keywords:** Acceleration, Visual perception, Time-to-collision estimation, Pedestrian-vehicle interaction, Traffic safety

## Abstract

When a pedestrian intends to cross the street, it is essential for safe mobility to correctly estimate the arrival time (time-to-collision, TTC) of an approaching vehicle. However, visual perception of acceleration is rather imprecise. Previous studies consistently showed that humans (mostly) disregard acceleration, but judge the TTC for an object as if it were traveling at constant speed (first-order estimation), which is associated with overestimated TTCs for positively accelerating objects. In a traffic context, such TTC overestimation could motivate pedestrians to cross in front of an approaching vehicle, although the time remaining is not sufficiently long. Can a simple acceleration signal help improve visual TTC estimation for accelerating objects? The present study investigated whether a signal that only indicates whether a vehicle is accelerating or not can remove the first-order pattern of overestimated TTCs. In a virtual reality simulation, 26 participants estimated the TTC of vehicles that approached with constant velocity or accelerated, from the perspective of a pedestrian at the curb. In half of the experimental blocks, a light band on the windshield illuminated whenever the vehicle accelerated but remained deactivated when the vehicle travelled at a constant speed. In the other blocks, the light band never illuminated, regardless of whether or not the vehicle accelerated. Participants were informed about the light band function in each block. Without acceleration signal, the estimated TTCs for the accelerating vehicles were consistent with an erroneous first-order approximation. In blocks with acceleration signal, participants substantially changed their estimation strategy, so that TTC overestimations for accelerating vehicles were reduced. Our data suggest that a binary acceleration signal helps pedestrians to effectively reduce the TTC overestimation for accelerating vehicles and could therefore increase pedestrian safety.

## 1 Introduction

People regularly interact with moving objects in their environment where actions need to be precisely timed. For example, a volleyball player must accurately anticipate the motion of the falling ball in order to hit it at the right moment. In road traffic, the goal is to avoid colliding with other road users. Pedestrians intending to cross a busy road, for instance, should estimate the time it takes for an approaching vehicle to reach their position (time-to-collision, TTC) to make a road-crossing decision, because they can only cross the road safely if the TTC of the approaching vehicle is longer than the time needed for crossing. If their TTC estimations are however inaccurate, they may decide to cross the street before the approaching vehicle, even though the remaining time is not long enough. Clearly, such estimation errors could jeopardize pedestrian safety. Because pedestrians are not protected by, for example, a vehicle chassis, pedestrian collisions are more likely associated with severe injuries or death, so that they are considered vulnerable road users. The high numbers of >8000 pedestrians killed in traffic accidents in Europe in 2018 [1] and of the 400,000 pedestrians killed in traffic accidents worldwide each year [2] underscore this vulnerability of this specific group of road users.

Previous research has shown that people commit significant estimation errors in interaction with accelerating objects (e.g., Refs. [[3], [4], [5], [6], [7], [8], [9], [10], [11], [12], [13]]), suggesting that pedestrians’ perception is biased in a safety-threatening fashion. Therefore, the goal of this study was to investigate whether a simple visual signal that binarily indicates that an approaching vehicle is accelerating can improve TTC estimation so that overestimation is reduced, thereby increasing traffic safety.

### 1.1 Limitations in the visual perception of acceleration

In mathematical terms, the instantaneous acceleration *a* of an object equals the instantaneous rate of change in velocity *v* over time, i.e., the second derivate with respect to time of the object distance *D* at a given point in time *t*. Based on these basic motion principles, the actual TTC of an object traveling with a constant acceleration rate *a* at time point *t* is determined by the object's instantaneous distance *D(t)*, instantaneous velocity *v(t),* and acceleration rate *a*, as precisely described in the following Eq. (1):

1 $$TTC\left(t\right)=\left\{ \begin{array}{c} \frac{-v\left(t\right)+\sqrt{2a∙D\left(t\right)+{\left(v\left(t\right)\right)\;}^{2}}}{a\;},a\neq 0\\ \;\\ \frac{D\left(t\right)}{v\left(t\right)},a=0 \end{array}\right.$$

Thus, to accurately predict the TTC of an accelerating object (*a* ≠ 0), both velocity (*first-order motion information*) and acceleration (*second-order motion information*) must be considered. The successful consideration of both velocity and acceleration represents a so-called *second-order estimation* and permits perfect TTC estimation for objects that accelerate at a constant rate. However, if an object approaches at a constant velocity (*a* = 0), the TTC is specified by the simple ratio between the instantaneous distance and the constant velocity (see Eq. (1)).

The erroneous visual TTC estimations for accelerating objects consistently reported in the literature (e.g., Refs. [3,[6], [7], [8]]), including approaching vehicles in a traffic context [[12], [13], [14]], follow a specific pattern that indicates a *first-order TTC estimation* (termed *TTC1* in the following) (e.g., Refs. [8,15]). That is, observers estimate the TTC for an accelerating object as if it moved at a constant velocity. The first-order estimate of TTC is the ratio between the instantaneous distance and the instantaneous speed at the moment of estimation, *TTC1*(*t*) *= D(t)/v(t)*. Observers using this strategy will estimate that a positively accelerating object requires more time to travel to a given position in space than it is actually the case. This dynamic relationship between *TTC1*(*t*) and the actual TTC of the accelerating vehicle, *TTC(t)* (Eq. (1)Eq., *a* ≠ 0)*,* depends on *TT the actualC(t)*, acceleration *a*, and velocity *v(t)* at the moment of estimation,

2 $$TTC1\left(t\right)=\frac{D\left(t\right)\;}{v\left(t\right)}=TTC\left(t\right)+\frac{a∙{\left(TTC\left(t\right)\right)}^{2}\;}{2v\left(t\right)\;}\text{.}$$

In the case of positively accelerating approaching objects (*a* > 0, *TTC*(*t*) > 0, *v*(*t*) > 0), the second term on the right-hand side of Eq. (2) is positive and specifies the extent of TTC overestimation, which grows with increasing actual TTC and acceleration, but decreases with the velocity at the moment of estimation. For objects approaching at a constant velocity (exclusive first-order motion, *a* = 0), in contrast, no estimation error is expected since the second term on the right-hand side becomes 0, so that *TTC1*(*t*) equals *TTC*(*t*). Previous studies consistently showed that humans base their TTC estimation for accelerating objects predominantly on first-order information in the context of a large range of different stimuli, such as a falling ball [8], simple geometrical objects directly approaching the observer [6,9], more meaningful objects in a traffic context approaching the observer directly or passing the observer [12,16], as well as simple objects moving towards a point in space on the frontoparallel plane [3,4,17]. A first-order estimation strategy for accelerating objects is indicated if the estimated TTCs for these objects match those for objects moving at constant velocity (exclusive first-order motion), provided that the instantaneous velocity and distance are the same. The failure to consider acceleration during TTC estimation may be related to the rather low sensitivity of our visual system to detect speed changes [[18], [19], [20], [21], [22], [23], [24], [25], [26]]. The approximate threshold for detecting acceleration is about an approximate 15–25 % change of the initial speed of an object [[26], [27], [28]].

In a traffic context, the TTC overestimation resulting from first-order estimation for positively accelerating objects might endanger road safety. If pedestrians estimate the arrival of an approaching vehicle to be much later than it is actually the case, they are likely to cross too close in front of that vehicle. As most accidents involving pedestrians occur in urban areas, most of them during road crossings [29,30], such urban road crossing situations seem to be particularly relevant.

### 1.2 How can errors in visual TTC estimation be reduced?

In contrast to the brake lights, which inform road users about a deceleration of a vehicle, there are no signals explicitly communicating the accelerating state of a vehicle that could be used as source of information during TTC estimation - at least not in the visual domain. In the auditory domain, on the contrary, there is indeed a signal conveying acceleration information of a vehicle: the vehicle sound. The sound of a vehicle with internal combustion engine is highly effective to improve audiovisual TTC estimations for accelerating vehicles, as we showed in previous studies [11,12]. In a virtual traffic environment, participants stood at the curb like pedestrians intending to cross the road, and were approached by vehicles that either travelled at a constant velocity or accelerated. In one of the studies [12], the vehicles were always visible, but only in half of the trials, the sound of an internal combustion engine vehicle was presented in addition to the visual information. Without vehicle sound, the visual TTC estimations for the accelerating vehicles showed the expected first-order pattern. This indicates that participants did not adequately consider acceleration information. With added sound, however, participants estimated the TTC for accelerating vehicles significantly more accurately, suggesting that thier TTC estimation was not solely based on first-order information but that second-order motion information was considered as well. Although it is not yet clear how exactly the sound of a vehicle with internal combustion engine conveyed the acceleration, it proved to be a useful source of information for pedestrians, promoting the TTC estimation accuracy.

However, the number vehicles with internal combustion engine will diminish, while the number of quieter (electric) vehicles will (further) increase in urban areas. This mobility transition presumably endangers pedestrian safety as the sounds emitted by electric vehicles are less effective in the communication of acceleration to road users. In fact, another recent study showed that the sound of an electric vehicle did not significantly facilitate the consideration of second-order information to the extent that the sound of an internal combustion vehicle did [11]. For accelerating electric vehicles, pedestrians in a virtual urban traffic scene increasingly overestimated the TTC at longer actual TTCs, higher acceleration rates and lower velocities. Accordingly, this pattern resembles that of a first-order estimation and predicts potentially dangerous estimation errors of pedestrians in interaction with accelerating electric vehicles.

Finally, we investigated whether feedback training could help to reduce pedestrians' TTC estimation errors for visually presented accelerating vehicles due to the consideration of first-order information only in a recent study [14]. With a similar scene setup as in Refs. [11,12], we measured estimated TTCs for constant-speed and accelerated approaches in three blocks, of which the second block provided trial-by-trial feedback about the TTC estimation accuracy. Although participants modified their TTC judgments during and after the feedback block, they did not manage to distinguish between accelerated and constant-speed approaches. Hence, the feedback training did not significantly promote the consideration of acceleration information, suggesting that it is not an effective solution for rectifying pedestrians' inaccurate TTC estimations for accelerating vehicles.

The present study investigated the effectiveness of a simple visual signal indicating the state of acceleration as a potential countermeasure against TTC overestimation for positively accelerating vehicles. In one of the experimental conditions, an acceleration light at the front of the simulated approaching vehicle binarily indicated the vehicle's state of acceleration (accelerating yes/no) to other road users. The subject of this study is relevant for both practical application and basic research. If a simple visual acceleration signal reduces TTC overestimation (first-order pattern), the implementation of such a signal could constitute the solution to simultaneously reduce noise emissions in urban areas while maintaining pedestrian safety. For basic research, the potential effect of a binary acceleration signal is related to the question whether explicit knowledge about the vehicle's state of acceleration (*a* = 0 versus *a* > 0) is sufficient to reduce the systematic TTC estimation errors for accelerating objects. The acceleration signal would provide distinctive information about the acceleration state, circumventing the need to detect the acceleration from the visual flow field. But is it sufficient for a pedestrian to know whether an object is accelerating or not in order to successfully reduce the first-order patterns and the associated overestimated TTCs?

We expected that a visual signal simply indicating whether a vehicle accelerates (acceleration signal on), or travels at a constant velocity (acceleration signal off) would reduce the first-order approximation in pedestrians’ TTC judgments, which is typically observed when only visual information about the approaching object is available. Put differently, we expected that the visual acceleration signal would mitigate potentially dangerous TTC overestimations in a (virtual) road crossing scenario.

## 2 Methods

### 2.1 Prediction-motion task

**Fig. 1:**
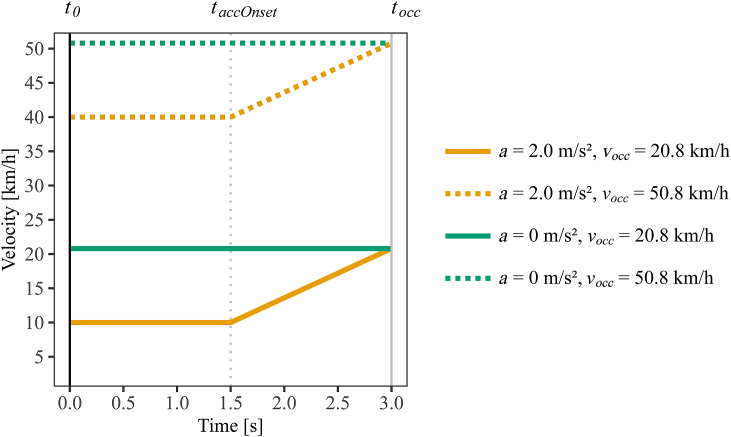
Velocity profiles of the four different approach conditions across the time course of a trial. At the beginning of the trial (*t*_*0*_), the vehicle approached at a constant velocity and subsequently either started to accelerate at *t*_*accOnset*_ or continued to travel at the same constant velocity. After the vehicle's occlusion (*t*_*occ*_), participants indicated the anticipated moment of the vehicle's arrival at their position. Orange lines: accelerating vehicle approaches (*a* = 2.0 m/s^2^). Green lines: constant-velocity approaches (*a* = 0 m/s^2^). Dotted colored lines: high velocity at occlusion (*v*_*occ*_ *=* 50.8 km/h). Solid colored lines: low velocity at occlusion (*v*_*occ*_ = 20.8 km/h). (For interpretation of the references to color in this figure legend, the reader is referred to the Web version of this article.)

Participants estimated the TTC of vehicles approaching in a visual virtual reality (VR) simulation in a prediction-motion task [31]. To start a trial, participants pressed a button on a controller, which triggered the vehicle motion (time point *t*_*0*_). In the simulation, the vehicle first approached the participants along a road at a constant velocity for 1.5 s and subsequently either accelerated with a = 2.0 m/s^2^ for 1.5 s or continued to travel with the same constant velocity for another 1.5 s (Fig. 1). After 3.0 s of presentation, the vehicle disappeared from the display as if passing behind an invisible occluder (occlusion time *t*_*occ*_). Participants were instructed to imagine that the vehicle continued to move exactly as it had before occlusion, i.e., they were to assume that the vehicle continued to approach with the exact same constant velocity or acceleration as they had last seen it. At the moment the participants thought that the vehicle would have arrived with its front at their position, they were instructed to pull a trigger on the controller, which ended the trial. The time between the occlusion and the activation of the trigger was taken as the estimated TTC (*TTC*_*est*_) in the given trial. Participants did not receive any feedback on the accuracy of their TTC estimations. Note that although the vehicle was not on a direct collision course with the virtual observer, we use the term time-to-collision for simplicity.

**Fig. 2:**
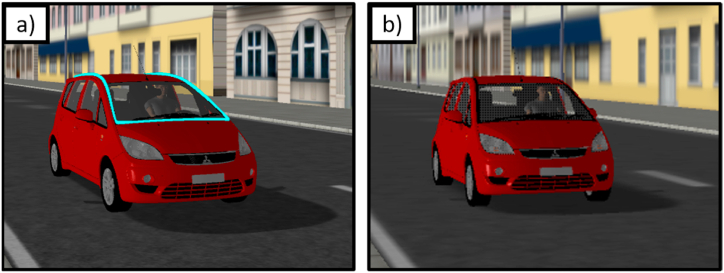
Simulated vehicle with *a*) activated light band around the windshield, and *b*) deactived light band.

Central to the experiment was a cyan light band around the windshield, which illuminated when the vehicle was accelerating in half of the experimental blocks, explicitly signaling the state of acceleration to the participants (Fig. 2). It was modeled after an external human-machine interface designed in the scope of the European interACT project [32] that has been previously used/adopted to communicate different content to road users, such as whether a vehicle was driving autonomously or manually controlled (e.g., Refs. [[33], [34], [35], [36]]). In blocks with the acceleration signal, the light band illuminated at acceleration onset (*t*_*accOnset*_) until occlusion (*t*_*occ*_) (Fig. 2a), but did not illuminate when the vehicle travelled at a constant velocity (Fig. 2b). In the other half of the experimental blocks, however, the light band never illuminated, neither for accelerated nor for constant-velocity approaches (Fig. 2b).

### 2.2 VR traffic scenario

**Fig. 3:**
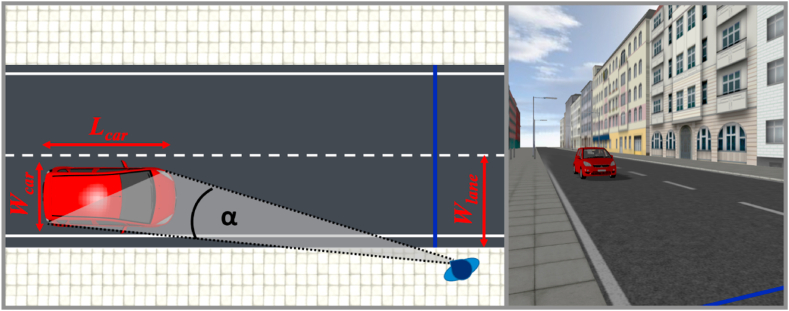
Layout out the depicted scene. Left: Bird's eye view of the participant at the curb observing the approaching car (dimensions: *L*_*car*_ = 3.95 m, *W*_*car*_ = 1.68 m) on the near lane (*W*_*lane*_ = 4.965 m) with the viewing angle α. Right: Participant's perspective of the scene as depicted in the head-mounted display.

The virtual traffic scenario showed a two-lane asphalt road (see Fig. 3). One lane of the road was 4.965 m wide (*W*_*lane*_ Fig. 3). Along the road were a pedestrian walkway and an adjacent gapless front of buildings, reminiscent of a typical urban environment. The street was simulated according to a model of Eislebener Straβe in Berlin, Germany. The virtual position of the participants was on the right pedestrian walkway in the direction of vehicle approach, 50 cm away from the curb. 50 cm to the left of the participants was a blue line extending horizontally from the left curb across the street to the right curb. The line helped the participants orient themselves in the virtual environment. The vehicle that approached the participants on the near lane in the simulation was modeled after a red Mitsubishi Colt (dimensions: *L*_*car*_ = 3.95 m, *W*_*car*_ = 1.68 m, *H*_*car*_ = 1.52 m, Fig. 3). The distance between the midpoint of the vehicle and the right curb was 1.625 m. Besides this vehicle, there were no other vehicles in the traffic scene. The driver of the vehicle was a male avatar with a neutral facial expression.

The traffic simulation was run by the VR software WorldViz Vizard 5 and was presented stereoscopically via an HTC Vive Pro head-mounted display with laser-based head-tracking (1440 × 1600 pixels per eye, 90 Hz frame refresh rate, 110° field of view, animation update rate 40 Hz). The tracking system translated the participants (head) movements into the virtual environment, so that the field of view of the virtual camera changed according to the real head movements. This allowed the participants to explore the virtual street scene with head movements and follow the vehicle's trajectories with their gaze. Note that the observers were able to turn their heads but they were instructed to stand at a fixed position in the laboratory room. Using an HTC Vive Pro manual controller, participants started each trial of the experiment at their own pace and performed the TTC estimation task.

### 2.3 Participants

As preregistered (As Predicted #76131), we recruited a sample consisting of *n* = 26 participants because we wanted the present and our previous study [12] to be comparable in terms of sample size as we expected a similar effect. In our previous study, we had collected data of 25 participants but in the present study, an even number of participants was needed to counterbalance the order of the acceleration signal conditions. The 26 participants (*M*_*age*_ = 31.42 years, *SD*_*age*_ = 15.09 years), of which 18 were women and 8 men, completed the experimental procedure. All had normal far visual acuity or wore contact lenses (corrected-to-normal far visual acuity), and did not suffer from a seizure disorder. The experiment was conducted in accordance with the ethical principles of the Declaration of Helsinki and the Ethics Committee of the Institute of Psychology at Johannes Gutenberg University Mainz (approval number: 2019-JGU-psychEK-S011). All participants volunteered (some for course credit). After providing study information and explaining possible risks, they gave written informed consent before the experiment started. After the completion of the experiment, participants received information about the experimental hypotheses.

### 2.4 Experimental design

**Fig. 4:**
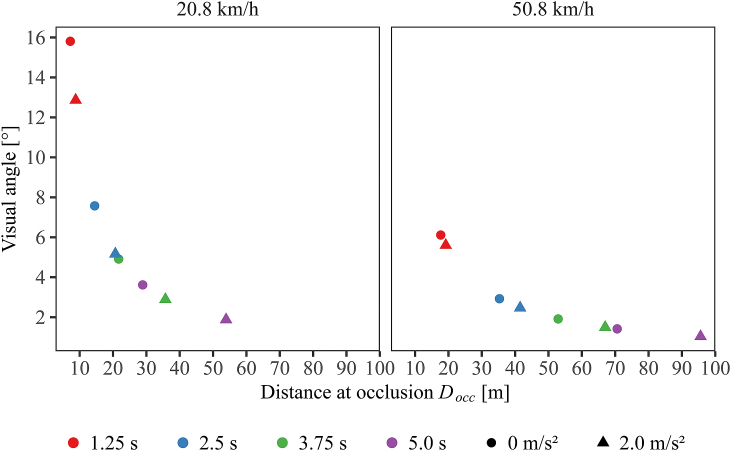
Optical size (i.e., the angle between the right vehicle front and left vehicle rear corner from the participant's perspective) as a function of distance at occlusion *D*_*occ*_, each for a velocity at occlusion of 20.8 km/h (left panel) and of 50.8 km/h (right panel). Color: actual TTC. Shape: acceleration rate. (For interpretation of the references to color in this figure legend, the reader is referred to the Web version of this article.)

All participants received all factorial combinations of four actual TTCs (1.25, 2.5, 3.75, 5.0 s), two acceleration rates (*a* = 0, 2.0 m/s^2^), two velocities at occlusion (*v*_*occ*_ *=* 20.8, 50.8 km/h), and two acceleration signal conditions (light band explicitly signaling acceleration, light band not signaling acceleration). For constant velocity approaches (*a* = 0 m/s^2^), the velocity was equal to *v*_*occ*_ during the entire presentation duration. For accelerated approaches (*a* = 2.0 m/s^2^, similar to our previous studies [11,12,14]), the vehicles initially travelled at a constant velocity of 10 km/h or 40 km/h before acceleration onset and reached 20.8 km/h or 50.8 km/h at occlusion, respectively. The vehicle distance at occlusion and the visual angle subtended by the vehicle (α in Fig. 3) covaried as a function of actual TTC, velocity at occlusion and acceleration rate. The variation of the visual angle as a function of distance is depicted in Fig. 4. The two acceleration signal conditions were varied in blocks. The order of the acceleration signal conditions was counterbalanced. That is, half of the participants started with a block where the light band illuminated when the vehicle was accelerating, but did not illuminate during constant-velocity approaches (*acceleration signal* condition); the other half started with a block where the light band was never illuminated, regardless of whether the vehicle accelerated or travelled at a constant velocity (*no acceleration signal* condition). All participants received each of the two acceleration signal conditions 5 times, resulting in 10 experimental blocks. Within each of the experimental blocks, all combinations of the factors actual TTC, acceleration rate and velocity at occlusion were presented twice and in random order. Across the experiment, each participant received each of the 4 (actual TTC) × 2 (acceleration rate) × 2 (velocity at occlusion) × 2 (with/without acceleration signal) factorial combinations 10 times, resulting in a total of 320 trials per participant.

### 2.5 Procedure

At the beginning of the session, all participants received information about the experimental structure and task during the upcoming experiment, completed demographical questions, and passed a visual acuity test (Landolt ring optotype chart [37], required visual acuity >1.0) and a stereoscopic vision test (Titmus-test [38], required were at least 6 correct responses for the 9 presented binocular disparities of 800, 400, 200, 140, 100, 80, 60, 50 and 40 s of arc). The experimenter measured the participants’ interpupillary distance to adjust the two displays of the HTC Vive Pro accordingly. Prior to testing and after each of the 10 experimental blocks, participants rated their feeling of motion-sickness on the Fast Motion Sickness Scale [39] to detect potential symptoms of motion sickness. The scale ranged from 0 – “not sick at all” to 20 – “frank sickness”. Participants completed two blocks of 10 trainings trials each to get familiar with the virtual environment and the experimental task. One of those blocks represented the *no acceleration signal* condition, the other one represented the *acceleration signal* condition. Participants were informed about the light band function in each training block and experimental block. The experimenter proposed to take a break after each experimental block but asked participants to rest for at least 10 min after half of the experimental blocks. The experiment ended as participants completed a final questionnaire regarding their personal driving and experimental experience, and lasted for approximately 60 min.

## 3 Results

We excluded extreme data points in each combination of participant, actual TTC, acceleration rate, velocity and acceleration signal condition according to a Tukey criterion [40]. 74 trials (0.89 %) of the total number of 8320 trials were excluded from subsequent analyses because they were three interquartile ranges below the first or above the third quartile, so that 8246 trials remained for the analyses of the mean estimated TTCs (section 3.1) and estimation precision (section 3.2).

### 3.1 Mean estimated TTCs

**Fig. 5:**
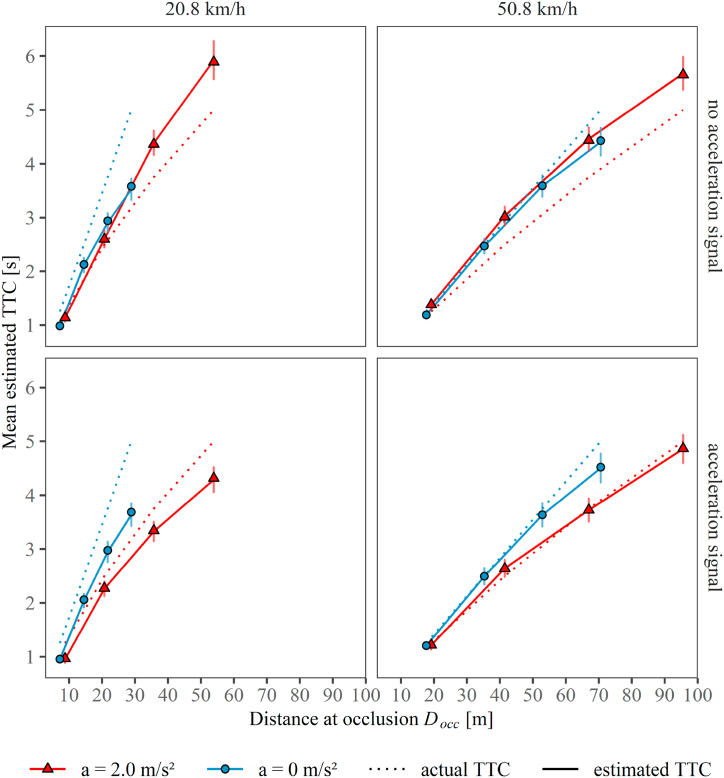
Mean estimated TTC for accelerating and constant-speed approaches as a function of distance at occlusion *D*_*occ*_ in blocks without (top row) and with acceleration signal (bottom row), at both velocities at occlusion *v*_*occ*_ (left column: 20.8 km/h, right column: 50.8 km/h). Red triangles: accelerating approaches. Blue circles: constant-velocity approaches. Actual TTCs (dotted lines) are depicted as reference to estimated TTCs (solid lines). Error bars indicate ±1 SE of the individual means. (For interpretation of the references to color in this figure legend, the reader is referred to the Web version of this article.)

**Fig. A1:**
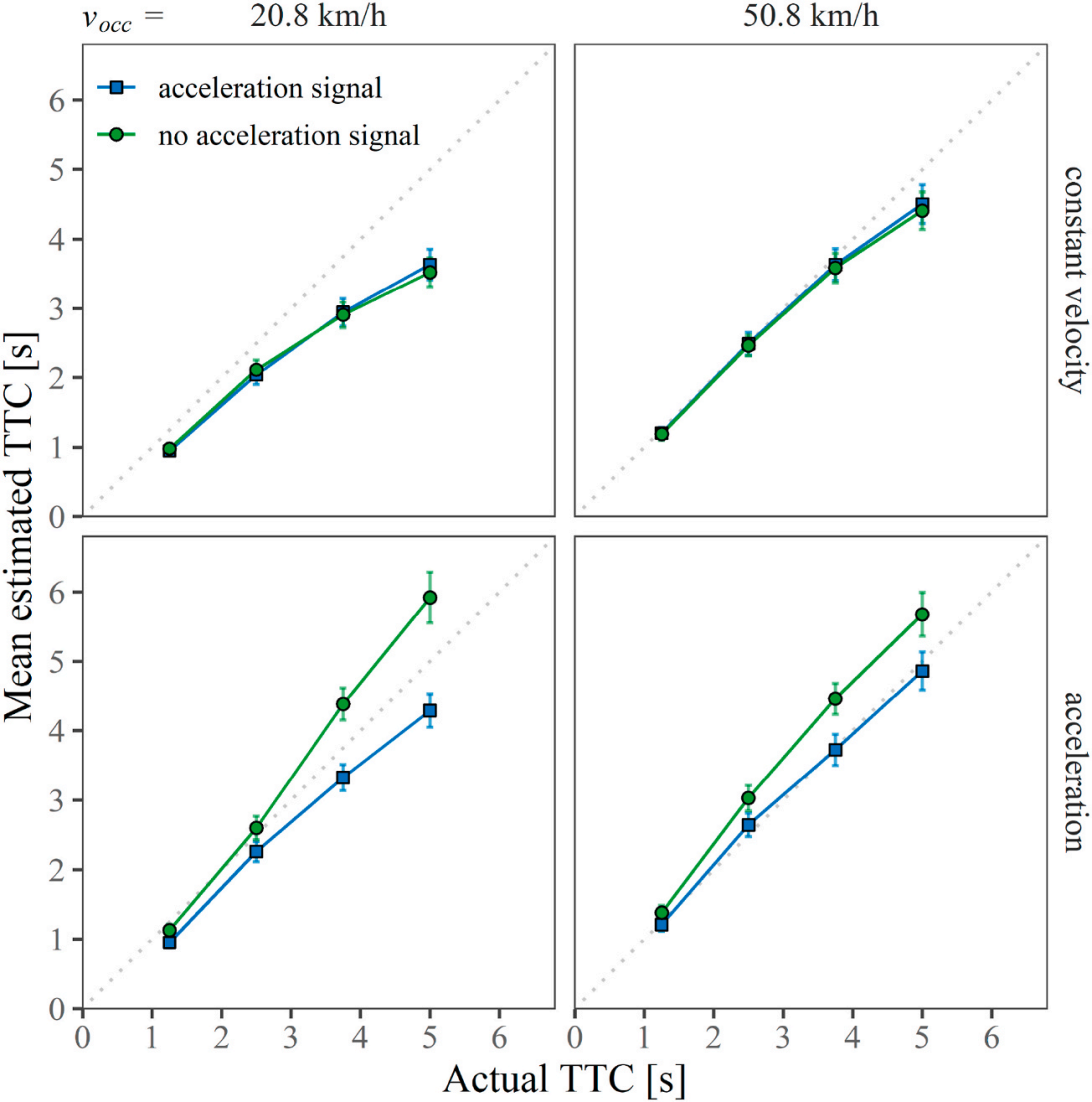
Mean estimated TTC for constant-velocity (upper row) and accelerating vehicle approaches as a function of actual TTC and velocity at occlusion *v*_*occ*_(left column: 20.8 km/h; right column: 50.8 km/h). Blue squares: visual signal (light band) indicated acceleration. Green circles: no visual acceleration signal was presented. Dotted grey line: perfectly accurate TTC estimation. Error bars indicate ±1 SE of the inidvidual means.

We aggregated the data per combination of participant and experimental condition to calculate the mean estimated TTCs. The mean estimated TTCs averaged across participants are illustrated as a function of the distance of the vehicle at occlusion *D*_*occ*_ in Fig. 5. To evaluate the extent to which the TTC estimations for the accelerating approaches are indeed compatible with a first-order estimation (instantaneous distance divided by instantaneous velocity, see Introduction) when no explicit visual acceleration signal is provided, they need to be compared with the estimated TTCs for constant-velocity approaches at identical distance at occlusion *D*_*occ*_ and identical velocity at occlusion *v*_*occ*_. As can be seen in the top row in Fig. 5, the mean estimated TTCs for accelerating approaches (red triangles) aligned approximately on the same function of *D*_*occ*_ as those for the constant-velocity approaches (blue circles) at the same *v*_*occ*_ (columns). Thus, the descriptive data clearly indicated that in blocks without acceleration signal, the estimated TTCs for accelerating vehicles were compatible with a first-order estimation strategy, where velocity but not acceleration information is considered. This resulted in TTC overestimation for the accelerating vehicles that increased with *D*_*occ*_*,* and thus with the actual TTC (red solid lines in Fig. 5; see also Appendix Fig. A1). In contrast, in blocks with acceleration signal (bottom row in Fig. 5), the functions relating the mean estimated TTCs and *D*_*occ*_ differed between accelerating and constant-velocity approaches. At each distance at occlusion, shorter TTCs were estimated for the accelerating compared to the constant-velocity approaches with the same velocity at occlusion, suggesting that the participants correctly considered that a positively accelerating vehicle needs less time to travel from a given position at occlusion than a vehicle traveling at a constant speed that was equal to the speed at occlusion of the accelerating vehicle. All in all, the acceleration signal shortened the mean estimated TTCs for accelerating vehicles, particularly at longer actual TTCs, so that the typical first-order pattern for accelerating vehicles (i.e., TTC overestimation increasing with the actual TTC) observed without acceleration signal was largely removed. Hence, the acceleration signal mitigated TTC overestimations, as expected.

**Fig. 6:**
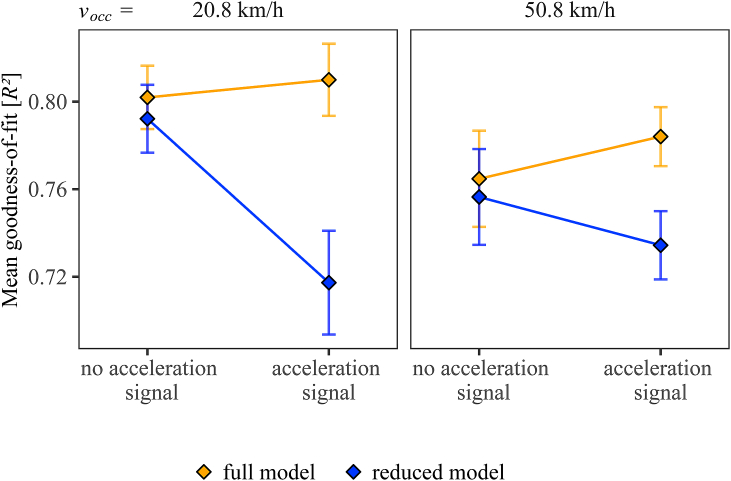
Mean goodness-of-fit (*R*^2^) as a function of acceleration signal condition, model type, and velocity at occlusion *v*_*occ*_ (left panel: 20.8 km/h, right panel: 50.8 km/h). Orange symbols: full model with separate functions fitted for accelerated and constant-velocity approaches. Blue symbols: reduced model with joint fit for accelerated and constant-velocity approaches. Error bars indicate ±1 SE of the individual means. (For interpretation of the references to color in this figure legend, the reader is referred to the Web version of this article.)

We hypothesized that the acceleration signal would substantially change the estimation strategy for accelerating vehicles in such a way that a first-order pattern and thus TTC overestimation would be reduced. In contrast to the condition without the acceleration signal, we thus expected that the TTC estimation strategy for accelerating vehicles with acceleration signal would differ from that for constant-velocity approaches with exclusive first-order motion. In general, the TTC estimation strategy can be defined by the following compressive function Eq. (3), which effectively describes the relation between the presented distance at occlusion (*D*_*occ*_) and the TTC estimations (*TTC*_*est*_) evident in Fig. 5,

3 $${TTC}_{est}=m\times D_{occ}^{k}\text{,}$$

where values of the exponent *k* < 1.0 of the exponent *k* represent a compressive relation between *D*_*occ*_ and *TTC*_*est*_. This function was fitted to the TTC estimates separately for each combination of participant, velocity at occlusion and acceleration signal condition, using the R-function *nls()*. In each of these combinations, we compared the goodness-of-fit of two nonlinear regression models statistically. In the *full* model, two separate functions (Eq. (3)) were fitted for accelerated and constant-velocity approaches, i.e., the estimated parameters $\hat{m}$ and $\hat{k}$ were allowed to differ between *a* = 0 and *a* = 2.0 m/s^2^. In the *reduced* model, a single function was jointly fitted to all datapoints (for both *a* = 0 and *a* = 2.0 m/s^2^), i.e., only a single value of $\hat{m}$ and $\hat{k}$ was estimated. If participants applied the same (first-order) estimation strategy for accelerated and constant-velocity approaches, which would result in similar TTC estimations at each combination of *D*_*occ*_ and *v*_*occ*_ for *a* = 0 and *a* = 2.0 m/s^2^*,* then the goodness-of-fit of the reduced model would be similar to the goodness-of-fit of the full model. If, however, participants used different TTC estimation strategies for accelerated and constant-velocity approaches, which would result in different TTC estimations at each combination of *D*_*occ*_ and *v*_*occ*_ for *a* = 0 versus *a* = 2.0 m/s^2^*,* then the goodness-of-fit should be substantially higher for the full model than for the reduced model. To test this, we analyzed the individual goodness-of-fit, measured as *R*^*2*^, as a function of model type (full model versus reduced model), velocity at occlusion, and acceleration signal condition. Since *R*^*2*^ represents a proportion between 0.0 and 1.0, we first applied an arcsine-square root transformation and subsequently used the transformed *R*^*2*^-values to calculate a three-factorial (model type, acceleration signal condition, *v*_*occ*_) repeated-measures ANOVA. Since we hypothesized that participants applied a first-order approximation for accelerating vehicles without acceleration signal but substantially changed their estimation strategy for accelerating vehicles with acceleration signal, we expected a significant interaction between model type and acceleration signal condition. We used *p*-values of < 0.05 as cut-off for statistical significance. Fig. 6 shows the mean goodness-of-fit as a function of model type, acceleration signal condition, and *v*_*occ*_.

When no acceleration signal was presented, the functions relating the mean TTC estimations to *D*_*occ*_ at a given *v*_*occ*_ were similar for accelerating and constant-speed approaches in Fig. 5, suggesting a similar (first-order) estimation strategy. Compatible with this assumption, the mean *R*^2^-values of the reduced model (joint fit to data points for *a* = 0 and *a* = 2 m/s^2^, blue symbols in Fig. 6) showed only a small descriptive difference compared to the full model (separate fits, orange symbols in Fig. 6) when no acceleration signal was presented. In contrast, in the condition with acceleration signal, the full model (separate functions (Eq. (3)) fitted for accelerated and constant-velocity approaches) resulted in a substantially higher mean *R*^2^ than for the reduced model (joint fit). This is in line with the assumption that participants used a substantially different TTC estimation strategy for accelerating vehicles compared to constant-speed approaches when the acceleration signal was available, which was already indicated by the differences between the functions relating the mean TTC estimations to *D*_*occ*_ at a given *v*_*occ*_ for accelerating and constant-speed approaches in Fig. 5. In the rmANOVA (Table 1) the interaction between model type (reduced model with joint fit versus full model with separate fits) and acceleration signal condition was significant. This interaction effect confirmed that the TTC estimations could be better described by two different functions relating *D*_*occ*_ and the estimated TTC rather than by a single function when the acceleration signal was available, but less so when the acceleration signal was not available. This effect of the acceleration signal on the difference between the goodness-of-fit of the two models was significantly more pronounced for the lower velocity at occlusion than for the higher one (left vs. right panel in Fig. 6), confirmed by a significant acceleration signal × model type × *v*_*occ*_ interaction. Note that the difference between a first- and a second-order TTC estimation increases with decreasing velocity at occlusion (Eq. (2)). Hence, it was not surprising that the difference in TTC estimation strategy for constant-velocity (first-order motion) and accelerated (second-order motion) approaches in the condition with acceleration signal was particularly prominent at the lower velocity - as evident in a larger difference in goodness-of-fit between the reduced and the full model in the condition with acceleration signal at the lower *v*_*occ*_ than at the higher *v*_*occ*_. Two follow-up rmANOVAs conducted separately for the lower and the higher *v*_*occ*_ showed a significant acceleration signal × model type interaction at both the lower and the higher *v*_*occ*_, *F*(1, 25) = 25.06, *p* < 0.001 and *F*(1, 25) = 16.06, *p* < 0.001, respectively.

**Table 1:** Results of the rmANOVA on the mean goodness-of-fit (arcsine-square root transformed *R*^*2*^ values). Regression model type (reduced model woth joint fit versus full model with separate fits), acceleration signal condition, and velocity at occlusion (*v*_*occ*_) served as within-subjects factors. Displayed are numerator degrees of freedom (*df*_*Num*_), denominator degrees of freedom (*df*_*Den*_), *F*-values, *p*-values and partial *η*^*2*^ (*η*^*2*^_*p*_). Cohen's *d*_*z*_ -values are additionally reported.

	df_Num_	df_Den_	F	p	η^2^_p_	d_z_
Acceleration signal	1	25	2.43	0.132	0.09	0.31
Model type	1	25	33.92	**<0.001**	0.58	1.14
*v*_*occ*_	1	25	7.74	**0.010**	0.24	0.55
Acceleration signal × Model type	1	25	23.23	**<0.001**	0.48	0.95
Acceleration signal × *v*_*occ*_	1	25	1.71	0.202	0.06	0.26
Model type × *v*_*occ*_	1	25	11.05	**0.003**	0.31	0.65
Acceleration signal × Model type × *v*_*occ*_	1	25	18.13	**<0.001**	0.42	0.84

Note: Bold font indicates statistical significance (*p* < 0.05).

Taken together, the acceleration signal helped participants to adjust their TTC estimation strategy for accelerating vehicles versus vehicles traveling at a constant velocity, particularly at the lower velocity at occlusion. This confirms the pattern evident in Fig. 5, where in the upper row (without acceleration signal), the function relating estimated TTC and *D*_occ_ is virtually identical for constant-velocity and accelerating approaches (indicating first-order estimation for the accelerating approaches), while in the lower row (with acceleration signal), the functions for constant-velocity and accelerating approaches differ substantially.

### 3.2 Estimation precision

In the next step, we analyzed the estimation precision, that is, the intra-individual variation in TTC estimation per experimental condition and participant (i.e., the “variable error” in terms of [41]). Previous studies analyzed either the standard deviation (*SD*) or the coefficient of variation (i.e., SD divided by mean) (e.g., Refs. [[42], [43], [44], [45]]). However, both metrics have certain limitations in interpretation. With increasing actual TTC (and usually also with increasing mean estimated TTC), estimated TTCs become increasingly variable [43,46,47]. This is not surprising, because with longer actual TTC there is also “more time” and thus a higher chance for misestimation (e.g., Ref. [15]). Thus, if in one condition A, the mean estimated TTC observed for a given participant is longer than in another condition B, it is likely that the SD across trials will also be larger in condition A. The coefficient of variation compensates for this dependence of the intra-individual variability on the mean estimated TTC by dividing the SDs of the estimated TTCs by their mean. Thus, the SDs are interpreted relative to their mean estimated TTCs. Unconfounding the SDs from the mean estimated TTCs makes sense for the reason mentioned above, but the calculation of the coefficient of variation effectively assumes that the SDs of the estimated TTCs are proportional to the mean estimated TTC, with a slope of 1.0. However, if the slope of the function relating the SD of the estimated TTCs to the mean of the estimated TTCs is lower or higher than 1.0, then the expression of the SD relative to the means is inadequate because it “overcorrects” or “undercorrects”, respectively. We therefore used a regression model to analyze the intra-individual variability of the estimated TTCs in relation to their means. For each combination of participant and experimental condition, the mean estimated TTC across the repeated trial presentations (*M*_*estTTC*_) was used as a continuous predictor, in addition to the categorical (backward difference coded) predictors *acceleration signal condition* and *velocity at occlusion* (*v*_*occ*_). The dependent variable was the SD of the estimated TTCs across the repeated presentations of exactly the same stimulus to the same participant (*SD*_*estTTC*_). This allowed us to compare the slope of the increase of the SD with the mean estimated TTC between experimental conditions, circumventing the need for an a-priori assumption concerning a specific slope. The regression model was specified as a Linear Mixed-Effects Model (LMM) that considered the repeated-measures structure by including the factor *participant* as random intercept. Degrees of freedom were estimated according to Kenward-Roger [48]. The model was fitted separately to the data for accelerating and constant-velocity vehicle approaches.

**Fig. 7:**
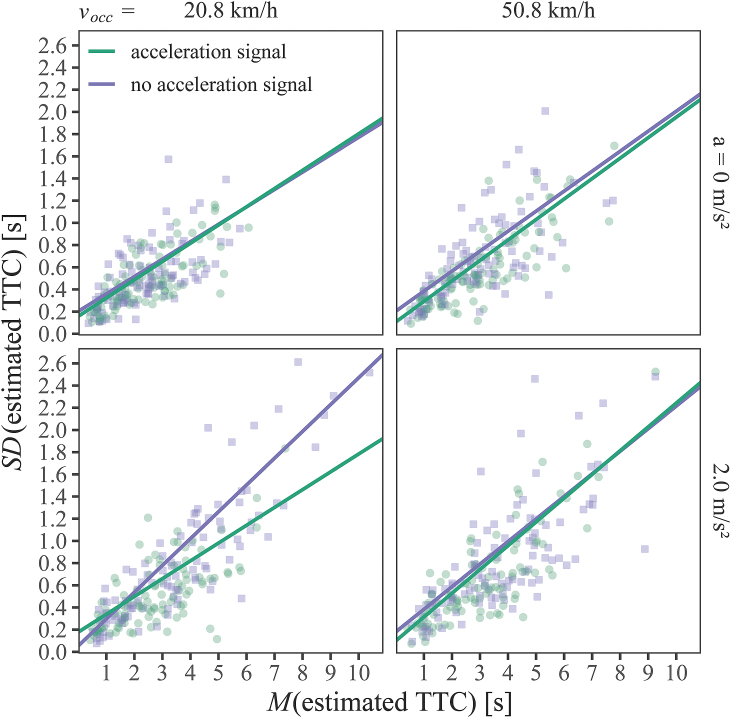
Standard deviations (*SD*) of the individual estimated TTCs as a function of the mean (*M*) estimated TTC, velocity at occlusion *v*_*occ*_ (left column: 20.8 km/h, right column: 50.8 km/h), acceleration rate *a* (top rows: 0 m/s^2^, bottom rows: 2.0 m/s^2^), and acceleration signal condition (green: acceleration signal condition, purple: condition without acceleration signal). The data points represent combinations of participant and presented TTC. Lines depict the LMM regression fits. (For interpretation of the references to color in this figure legend, the reader is referred to the Web version of this article.)

First, we analyzed the intra-individual SDs of the estimated TTCs (that is, estimation precision) for the *accelerating vehicles* in relation to the mean estimated TTCs. With its fixed and random effects, the regression model explained 74.93 % of the variance in the aggregated data for the accelerating vehicle approaches. The bottom row in Fig. 7 shows the SD of the estimated TTC as a function of their means. The estimated fixed effects parameters are depicted in Table 2. As expected, the SD significantly increased with increasing mean estimated TTC. However, Fig. 7 reveals that the slope of the regression line relating the SD to the mean of the estimated TTCs was substantially lower when the vehicle's acceleration was indicated by the light band than when it was not explicitly signaled (significant *M*_*estTTC*_ × *acceleration signal condition* interaction), at least at the lower velocity (left column), which was confirmed by the significant *M*_*estTTC*_ × *acceleration signal condition* × *v*_*occ*_ interaction in Table 2. Across the mean estimated TTCs, the model indicated a significantly lower SD in the presence of the acceleration signal than in its absence for the lower but not for the higher velocity at occlusion (interaction *acceleration signal condition* × *v*_*occ*_). All other regression coefficients were not significantly different from 0. Overall, the regression analysis suggests that the acceleration signal resulted in a higher estimation precision for accelerated approaches, i.e., reduced the variability in the individual TTC estimations, at least at the lower velocity at occlusion.

**Table 2:** Estimated fixed effects parameters of the regression model (LMM) used to analyze the intra-individual standard deviations of the estimated TTCs for the accelerating vehicles in relation to the mean estimated TTC (*M*_*estTTC*_), acceleration signal condition, and velocity at occlusion (*v*_*occ*_). Displayed are effect estimates (*β*), standard errors (*SE*), corrected degrees of freedom (*df*), *t*-, and *p*-values.

	β	SE	df	t	p
(Intercept)	0.06	0.04	62.09	1.55	0.127
*M*_*estTTC*_	0.19	0.01	406.94	26.03	**<0.001**
Acceleration signal condition	−0.02	0.05	384.31	0.45	0.654
*v*_*occ*_	0.02	0.05	383.81	0.50	0.618
*M*_*estTTC*_ × acceleration signal condition	0.04	0.01	385.85	2.64	**0.009**
*M*_*estTTC*_ × *v*_*occ*_	0.01	0.01	383.74	0.59	0.557
Acceleration signal condition × *v*_*occ*_	0.2	0.1	383.23	2.13	**0.034**
*M*_*estTTC*_ × acceleration signal condition × *v*_*occ*_	−0.09	0.03	383.45	3.48	**0.001**

Note: Bold font indicates statistical significance (*p* < 0.05).

The second LMM analyzed the SDs of the estimated TTCs (i.e., estimation precision) for the vehicles traveling at a constant velocity by the fixed effects *M*_*estTTC*_, acceleration signal condition and velocity at occlusion *v*_*occ*_, and explained 67.69 % of variance in the aggregated data for the constant-velocity approaches. As can be seen in the top row in Fig. 7 and Table 3, the estimation precision was predominantly affected by the mean estimated TTC, while neither the acceleration signal condition, the velocity nor their interaction had a significant effect. The SDs were significantly and positively associated with the mean estimated TTC, to a similar extent as for the accelerating vehicle approaches (*β*_*const*_ = 0.16, cf. *β*_*acc*_ *=* 0.19), showing the expected increase of the SDs of the estimated TTCs with increasing means.

**Table 3:** Estimated fixed effects parameters of the regression model (LMM) used to analyze the intra-individual standard deviations of the TTCs estimates for the vehicles at a constant velocity in relation to the mean estimated TTC (M_estTTC_), acceleration signal condition and velocity at occlusion (*v*_*occ*_). Displayed are effect estimates (*β*), standard errors (*SE*), corrected degrees of freedom (*df*), *t*-, and *p*-values.

	β	SE	df	t	p
(Intercept)	0.12	0.03	67.88	4.07	**<0.001**
*M*_*estTTC*_	0.16	0.01	407.53	21.40	**<0.001**
Acceleration signal condition	0.07	0.04	383.39	1.83	0.068
*v*_*occ*_	−0.02	0.04	384.24	0.63	0.529
M_estTTC_ × acceleration signal condition	−0.01	0.01	383.50	0.46	0.649
*M*_*estTTC*_ × *v*_*occ*_	0.02	0.01	385.64	1.66	0.098
Acceleration signal condition × *v*_*occ*_	0.05	0.08	383.17	0.68	0.497
*M*_*estTTC*_ × acceleration signal condition × *v*_*occ*_	0	0.03	383.20	0.13	0.898

Note: Bold font indicates statistical significance (*p* < 0.05).

## 4 Discussion

In this study, we investigated the effect of a binary visual acceleration signal at the front of an approaching vehicle on TTC judgments. From the perspective of a pedestrian, participants estimated the TTC for accelerated and constant-velocity vehicle approaches in a prediction-motion paradigm. In half of the experimental blocks, a light band around the vehicle's windshield illuminated during acceleration and remained deactivated when the vehicle moved at a constant velocity. In the other half of the blocks, the light band never illuminated, and thus provided no explicit information concerning the state of acceleration. As expected, in blocks without visual acceleration signal, participants performed a first-order approximation for accelerating vehicles, that is, the mean estimated TTCs for the accelerating approaches followed a similar function of *D*_*occ*_ as those for constant-velocity approaches (Fig. 5). This indicates that participants largely disregarded the presented acceleration, consistent with previous studies (e.g., Refs. [8,12]). Because the TTC overestimation resulting from this first-order estimation pattern might be associated with risky road crossing decisions, our data thus supported the assumption that pedestrian safety might be threatened in interaction with accelerating vehicles – at least when the vehicle sound is not audible [12]. In contrast, when the light band explicitly signaled the state of acceleration, the first-order TTC approximation pattern (TTC overestimation increasing with the presented TTC at occlusion) for accelerating vehicles was strongly reduced; the functions relating the mean estimated TTCs and *D*_*occ*_ in Fig. 5 now differed between accelerating and constant-velocity approaches. Hence, the acceleration signal helped participants to adjust their TTC estimation strategy for accelerating vehicles, resulting in reduced TTC overestimations, which was particularly pronounced at the lower velocity at occlusion. Furthermore, our data showed that for accelerating vehicles with a lower velocity at occlusion, the binary acceleration signal additionally reduced the intraindividual variation in the estimated TTCs, i.e., increased the estimation precision. Overall, a binary visual acceleration signal improved pedestrians' TTC estimation for accelerating vehicles in a safety-enhancing way.

### 4.1 How can the effect of a binary acceleration signal be explained?

The light band only provided information whether the vehicle was accelerating or not, but did neither indicate how strong the acceleration was, how fast the vehicle was traveling, nor how far the vehicle distance was. Still, participants relied on the acceleration signal as source of information, so that it substantially changed their TTC estimation strategies. But how did the binary information *that* the approaching vehicle accelerates modify the TTC estimation process? This is certainly a question that we cannot answer conclusively based on our data. However, the following theoretical considerations will point the way for the design of future studies. One possibility is that participants did not only consider first-order but also second-order (acceleration) information, allowing for more accurate TTC estimations. Thus, the acceleration signal might have enabled an “elaborate” change in estimation strategy, rather than promoting a simple safety strategy in form of an unspecific shortening of the estimated TTCs whenever the acceleration signal was present (e.g., by a constant duration or proportionally) (e.g., Ref. [44]).

In a previous article [12], we found that when the sound of an accelerating vehicle with internal combustion engine was presented in addition to the visual simulations, this mitigated the first-order TTC estimation observed in a visual-only condition. As one potential explanation, we discussed the – admittedly speculative – possibility that humans might in principle be able to perceive acceleration based on visual and/or auditory cues, but only factor it into their TTC estimations when they are explicitly aware that the approaching vehicle accelerates. In that sense, participants might only be able to use acceleration cues *after* they have detected that the approaching object is accelerating. In this line of reasoning, the vehicle sound might also have acted as a binary acceleration signal communicating to the pedestrians *whether or not* the approaching vehicle was accelerating, which in turn might have directed the participants’ attention to the relevant visual and/or auditory second-order information available in the traffic scene. However, the sound of an accelerating vehicle not solely signaled acceleration in a *qualitative*, binary manner, but in addition provided at least approximate *quantitative* information concerning the rate of acceleration in Ref. [12]. It was therefore not possible to decipher whether and how qualitative and quantitative second-order information were used for TTC estimations. In the present experiment, on the other hand, the acceleration signal definitively did not provide quantitative, but only qualitative information about acceleration. Thus, one potential explanation for the change in estimation strategy is that observers can immediately shift their attention towards visual second-order information as soon as the visual acceleration signal lights up, leaving sufficient time to integrate the motion signals for the estimation of vehicle acceleration, which can then be used during TTC estimation. If, however, no explicit acceleration signal is available, observers first have to detect the acceleration based on the visual flow field.

It has been proposed that observers perceive acceleration by comparing the relative difference in estimated velocity of an object at two time points (e.g., Ref. [27]). If so, the estimation of acceleration should be easier if it is based on two distinctly different velocity estimations (“snapshots”). For a constant acceleration rate as in the present experiment, the difference in velocity increases with the duration between two time points. Given that the acceleration signal reliably indicated the state of acceleration immediately at acceleration onset, participants were able – at least in principle – to use the entire acceleration presentation (1.5 s) to derive an estimated acceleration rate, for example, based on the relative difference in velocity between occlusion and acceleration onset. In contrast, without acceleration signal, participants likely either a) did not detect the vehicle was accelerating and thus did not consider acceleration at all that, or b) did not realize the acceleration only shortly before occlusion, so that the relative difference in velocity between the time of acceleration detection and occlusion was small, potentially resulting in an underestimation of the acceleration rate. In sum, the observed effect of the acceleration signal could have resulted due to an (earlier) attention shift towards acceleration information, and as a consequence, a relatively precise estimation of the acceleration rate that could be considered during TTC estimation.

An alternative explanation for the observed results is that participants did use acceleration information in their TTC estimations when the acceleration signal appeared, which was however not derived from the visual scene but was instead based on an a-priori assumption about the acceleration rate. Such an acceleration rate prior could be formed, for example, from everyday experiences with accelerating vehicles. Consistent with current theoretical perspectives on internal motion models, which could be shaped by, for example, experiencing the law of gravity [[49], [50], [51], [52]], the binary acceleration signal might have triggered the recall of previous traffic experiences, such that participants might have formed expectations about a typical acceleration rate. The study design does not allow us to decide whether the acceleration signal has either enabled a more accurate estimation of the acceleration rate or has triggered the recall of an acceleration rate prior. This is due to the fact that only one acceleration rate was implemented in the present study. For a critical test of the outlined two explanations, an experiment is needed in which at least two different acceleration rates are presented. If participants are able to actually estimate the acceleration rate from available visual cues when the visual signal indicates acceleration, the TTC estimation strategies should differ between the presented acceleration rates. If, however, the acceleration signal only elicits a prior about the acceleration rate, then the TTC estimation strategies should be quite similar for the different acceleration rates presented. A variation of the acceleration signal onset (independent of the acceleration onset) could be additionally helpful to test the hypothesis that observes estimate the acceleration rate based on the relative change in velocity between two time points.

Alternatively, the effect of the light band around the wind shield, which we implemented as an acceleration signal, could also be explained without assuming that participants explicitly factored the acceleration rate into their TTC judgments. Instead, it could be that the changes in the TTC estimation strategy arose from a higher considered constant velocity or a shorter considered distance at occlusion of the vehicle. In fact, the light band increased the contrast of the depicted approaching vehicle, and the alteration of contrast of an object changes the perceived velocity of the object [53,54]. Also, the perceived distance of an object decreases with increasing brightness contrast between object and background [55,56]. However, in the context of TTC estimation, a previous study suggests that participants do not consistently factor in the brightness contrast between object and background [57]. But even if the altered contrast due to the light band is not crucial in TTC judgments, the acceleration signal might still have had an impact on the perceived velocity and perceived distance during TTC estimation. As outlined before, a first-order estimation strategy, that is based on the velocity at occlusion, typically results in an overestimated TTC. This is because observers disregard that the vehicle will continue to increase its velocity between occlusion and its arrival at the pedestrian's position. However, the correction of this estimation error does not necessarily require an explicit consideration of the acceleration rate. Instead, the reduction of the TTC overestimation can effectively be achieved by assuming a higher (constant) velocity or a shorter distance than that presented at occlusion, i.e., by a modified first-order estimation strategy. Further research is needed to disentangle which mechanism(s) contribute(s) in what way to the effect of the acceleration signal, for instance, by presenting additional acceleration rates or by adding a control condition in which the light band is also activated in some trials of the constant-speed conditions. Nevertheless, this does not limit the conclusion that the light band as an acceleration signal effectively reduced the extent of TTC overestimation observed without explicit signal.

In addition to the outlined positive effect of the binary acceleration signal on estimation accuracy, pedestrians estimated the TTC for accelerating vehicles also with a significantly higher precision, i.e., lower variability than without it – at least at the lower velocity at occlusion. A higher precision could both be compatible with the hypothesis of “attention shift” or “internal prior”. In case that the acceleration signal immediately directs the observer's attention to the available second-order information, the observers have a relatively long time to estimate the acceleration, which might promote a more accurate but also a more precise acceleration estimate. However, if the acceleration of the vehicle is detected by observers only in some of the trials, or at a later point in time during the acceleration phase, as could be the case when no acceleration signal is provided, this could lead to higher trial-to-trial variability in the estimated acceleration rates and, as a result, in the estimated TTCs. On the other hand, if the acceleration signal triggers the use of an acceleration rate prior, the same prior would have been used on each trial on which the acceleration signal appeared, again reducing the trial-to-trial variability in the estimated TTCs. However, in our view the alternative explanation of the effect of the acceleration signal in terms of increased perceived velocity at occlusion or decreased perceived distance is difficult to reconcile with the effect of the acceleration signal on the estimation precision. If the acceleration signal altered the perception of velocity and/or distance, this would explain the reduced TTC overestimation for accelerating vehicles, but it is less evident how the trial-to-trial variability in the estimated TTCs should be reduced.

### 4.2 TTC estimation for constant-velocity approaches

For constant-velocity vehicle approaches, the acceleration signal never appeared. Hence, stimuli were identical in both acceleration signal blocks. It is thus not surprising that participants judged the TTC for vehicles approaching at a constant velocity on average similar in both signal blocks (Fig. 5, see also Appendix), suggesting that the knowledge that a vehicle travelled at a constant velocity did not substantially influence the TTC estimation accuracy. Regarding the estimation precision, the SDs increased as a function of the mean estimated TTCs, consistent with previous results [43,46,47]. Beyond that, neither the velocity nor the acceleration signal block had a significant effect on the estimation precision.

The mean estimated TTCs for constant-speed approaches showed characteristic biases. First, at each constant velocity, they were a compressive function of *D*_occ_ (Fig. 5), which we accounted for in the data analysis (Eq. (3)). Also, the mean estimated TTCs showed a stronger underestimation of the presented TTCs at the lower compared to the higher constant speed (Fig. 5). Put differently, at a given TTC, the mean estimated TTC was lower at the slower than at the faster speed, as shown in Fig. A1. Since at the slower constant velocity, the *D*_occ_ at a given TTC was smaller and the optical size was larger than at the faster speed, this result is compatible with a distance bias [58] or a size-arrival effect [[59], [60], [61], [62], [63]]. In this study, the two effects cannot be distinguished because the physical size of the vehicle did not change and thus distance and optical size at occlusion were perfectly correlated.

### 4.3 Limitations

As we have already outlined above, two noteworthy limitations of our study are 1) that we have only implemented a single acceleration level, and 2) that we cannot conclude about the potential mechanisms driving the effect of the binary acceleration signal (e.g., change in considered acceleration, velocity and/or distance). In addition, we acknowledge that pedestrians' real-world crossings might involve a complex and iterative interplay between perception and behavior, where an on-line control strategy might be used to adapt the behavior towards the vehicle [64]. In our investigation, we have aimed to contribute insights into a specific aspect of this complex process by focusing on TTC estimation as a perceptual measure because it plays a pivotal role for pedestrians [65]. From a pedestrians’ perspective, the TTC estimations are, in principle, an appropriate basis to make informed road-crossing decisions. To accurately assess TTC judgments, we have implemented an occlusion paradigm, which is a well-established method. However, it should be noted that pedestrians in real-world situations may assess TTCs to make an initial crossing decision but may subsequently apply, for instance, an on-line control strategy [64] to further adapt their behavior during the crossing itself. Furthermore, we have only simulated one specific traffic scenario, in which a single vehicle approached the pedestrian on a two-lane road from the left-hand side. However, real-world traffic scenarios may involve intersecting roads with multiple road-users approaching the pedestrian from different directions or with varying driving profiles. Since previous research showed that the presence of other road-users affects TTC estimations, even when irrelevant to the observer [66], the present findings should be extended in future experiments to capture the diversity of real-world environments.

### 4.4 Conclusions

Pedestrians tend to commit estimation errors in predicting the time-to-collision (TTC) of a vehicle approaching with positive acceleration. They do not adequately account for acceleration, but judge the TTC for an accelerating vehicle as if it moved at a constant speed (first-order approximation). As a result, they overestimate the TTC, which is associated with risky pedestrian behavior, i.e., unsafe crossings. In half of the experimental blocks in the present study, a light band around the windshield explicitly signaled whether or not the vehicle accelerated, while the accelerating state was not explicitly indicated in the other half of the blocks. Without the acceleration signal, the estimated TTCs for accelerating vehicles were compatible with a first-order estimation strategy, as expected. With the acceleration signal, however, participant adjusted their TTC estimation strategy for accelerating vehicles, resulting in reduced TTC overestimations. Thus, a simple binary acceleration signal promoted a significant change in estimation strategy. Furthermore, our data showed that the acceleration signal significantly reduced the intraindividual variation in estimated TTCs, i.e., increased the estimation precision, for accelerating vehicles with a relatively slow velocity at occlusion. From a practical perspective, a binary acceleration signal could help pedestrians to effectively reduce TTC overestimation for an approaching accelerating vehicle when deciding whether it is safe to cross the road in front of it or not.

## Funding

This research did not receive any specific grant from funding agencies in the public, commercial, or not-for-profit sectors.

## Data availability statement

All data are shared openly alongside the manuscript (Open Science Framework, https://osf.io/qnvyc/?view_only=71ecd919b66542eea59de370a8fbcd58). The analysis code is available upon request.

## CRediT authorship contribution statement

**Marlene Wessels:** Writing – review & editing, Writing – original draft, Visualization, Supervision, Software, Resources, Project administration, Methodology, Investigation, Formal analysis, Data curation, Conceptualization. **Daniel Oberfeld:** Writing – review & editing, Validation, Supervision, Resources, Project administration, Methodology, Formal analysis, Conceptualization.

## Declaration of competing interest

The authors declare that they have no known competing financial interests or personal relationships that could have appeared to influence the work reported in this paper.
